# Movement-specific keyboard playing for hand function in adolescents and young adults with acquired brain injury

**DOI:** 10.3389/fneur.2022.1062615

**Published:** 2023-01-09

**Authors:** Soo Ji Kim, Yoon-Kyum Shin, Eomhyeong Jeong, Sung-Rae Cho

**Affiliations:** ^1^Music Therapy Education, Graduate School of Education, Ewha Womans University, Seoul, Republic of Korea; ^2^Arts Education Therapy Institute, Ewha Womans University, Seoul, Republic of Korea; ^3^Department and Research Institute of Rehabilitation Medicine, Yonsei University College of Medicine, Seoul, Republic of Korea; ^4^Brain Korea 21 PLUS Project for Medical Science, Yonsei University College of Medicine, Seoul, Republic of Korea; ^5^Graduate Program of Biomedical Engineering, Yonsei University College of Medicine, Seoul, Republic of Korea; ^6^Rehabilitation Institute of Neuromuscular Disease, Yonsei University College of Medicine, Seoul, Republic of Korea

**Keywords:** keyboard playing, rehabilitation, hand function, adolescents, acquired brain injury

## Abstract

**Background:**

Patients with acquired brain injury (ABI) suffer from deficits in fine motor function in hands which affect independent self-care function in daily life. This study aimed to examine the effects of movement-specific keyboard playing for improved hand function in adolescents and young adults with ABI.

**Method:**

A total of 23 patients with ABI participated in this study. Twelve were assigned to the intervention group and eleven to the control group. The intervention group engaged in movement-specific keyboard playing three to four times a week for 3 weeks in addition to standard care, while the control group received only standard care.

**Results:**

The results of a mixed model of repeated measures ANOVA showed that the time effects were significant in the functional independence measure, key-pressing force, and most of the hand function tests measured. In terms of the interaction effect between group and time, a significant effect was found only in the checker-stacking task as a subtest of the Jebsen-Talyor Hand Function Test.

**Discussion:**

These results indicate that the specified movements required to play the keyboard may involve more precise and dexterous manipulation with hands and fingers. These results also suggest that movement-specific keyboard playing has potential in optimizing the intervention effect of keyboard playing while maximizing the benefits of music for motivating young patients with ABI.

## 1. Introduction

Deficits in fine motor control after brain injury affect self-care (e.g., feeding, washing, and dressing) and hand-motor tasks utilized in everyday life (1). For patients recovering from brain injury, the degree of improvement in such skills is documented to be less than that of the degree of improvement in gait function, and such deficits remain considerable compared to healthy control groups (2). Reduced strength and impaired coordination of the hand and finger muscles due to brain injury limit engagement in daily activity. A previous study found that the level of impairment was positively correlated with the degree of limitation in daily activities (1). Such limitation in younger individuals is a critical issue because they require more active and continuous engagement in school or the workplace. Young patients with stroke report limitations in keeping up with peers at school in the following areas: utilizing school facilities, engaging in activities that require fine motor function, and self-care skills (1).

Instrument playing-based rehabilitation has been repeatedly documented as an effective intervention for fine motor skills in individuals with brain injury (3). In particular, the keyboard has been used in various ways to engage users in repetitive and patterned movements for such rehabilitative training. During keyboard playing, involved individuals are asked to produce specific sounds, providing immediate and direct feedback on the executed movements and helping them plan subsequent movements. Such auditory-motor coupling was demonstrated with untrained healthy adults (4) and patients with chronic stroke (5). Through the coupling mechanism, neuroplastic changes were observed in terms of recovery of selective motor control by inhibiting the contralateral activation to compensate for the lesion on the affected side as well as facilitating the affected side, which was the primary foundation of instrument playing for rehabilitation.

Attempts to incorporate keyboard playing into rehabilitative interventions for proximal upper limb stability and distal fine motor skills have been promising. Previous studies have demonstrated that rehabilitative training based on keyboard playing increased the key-pressing force of adolescents and young adults who showed deficient fine motor control due to neurological impairment (6–9). Furthermore, as a part of the multi-instrument playing method, keyboard playing was integrated to induce finger individuation (10). Such key-pressing force during keyboard playing in patients with stroke was correlated with the ability to manipulate objects (11). Moreover, engagement in intensive keyboard playing was also found to lead to improvements in dexterous movements measured by the Box and Block Test (BBT) and the nine-hole pegboard test, which measures manual dexterity by requiring individuals to grasp small objects and manipulate them (transport or place into small holes) (12, 13).

Given the increased effort to develop interventions that use keyboard playing, it is necessary to investigate how such keyboard-playing tasks can be optimally constructed. A previous experimental study examined how the type of finger movement (e.g., individuated vs. sequential movements) and the adjustment of tempo differently elicited muscle activity (14), suggesting that the type of keyboard-playing task would be a critical agent for intervention outcomes. Although many previous studies have systematically ordered the levels of finger exercises, the determination of the task level tended to depend on the musical context (e.g., single note vs. the melodic sequence of the actual music). When documenting musical outcomes (e.g., the melody of a song played when a patient practiced finger movements) to increase patient motivation (15), it has been suggested to use more varied musical pieces. Task levels might be influenced by the difficulty of the music utilized (i.e., melody), yet such an approach might be rather limited and require the consideration of other variables (e.g., cognitive function to understand the musical notes or musical context, or complex patterns of finger movements to produce a specific melody).

Accordingly, this study utilized a constructed, movement-specific keyboard-playing intervention and investigated how it leads to functional outcomes in adolescents and young adults with acquired brain injury. As an attempt to extend a preliminary study (7), the current study more systematically examined the intervention by selecting and adjusting the level of tasks primarily based on the type of finger movement. This study also included a control group to examine how such keyboard playing differently affected patients' functional movements when movement-specific keyboard playing was applied in addition to standard rehabilitative training.

## 2. Materials and methods

### 2.1. Participants

All study procedures were approved by the Institutional Review Board (IRB No. 4-2012-0483). A total of 23 patients aged 13–29 years with acquired brain injury were recruited from a rehabilitation hospital from July 2013 to May 2018. We obtained written informed consent from each participant and their caregivers (for adolescents). The inclusion criteria for each participant were as follows: (1) acquired brain damage through cancer, traumatic brain injury, stroke, or other non-congenital causes and (2) impaired hand functions with respect to performing activities of daily living. The assessment of hand function was measured by the self-care subtest of functional independence measure (FIM), and each participant was screened with assistance from a helper in performing tasks (i.e., eating, grooming, bathing, dressing, and toileting). Detailed information about the participants is displayed in Table 1. Participants were randomly assigned to the experimental group or the control group. Participants assigned to the control group received only standard care, while participants assigned to the experimental group received movement-specific keyboard playing in addition to standard care. As standard care, both groups received physical therapy and occupational therapy five times per week during the treatment period.

**Table 1:** Demographic information of participants.

	**Intervention group (*n =* 12)**	**Control** **group (*n =*11)**
Sex, Male/Female	5/7	8/3
Age, yrs (*M* ±*SD*)	14.3 ± 5.7	15.5 ± 4.68
Affected hand, Lt. [*n* (%)]	7 (58.3)	8 (80.0)
Duration since onset, yrs (*M* ±*SD*)	5.0 ± 6.4	2.5 ± 4.4
**Diagnosis (** ***n*** **)**		
Traumatic brain injury	3	6
Stroke	6	4
Brain tumor	3	1

Lt, left.

### 2.2. Apparatus

**Figure 1:**
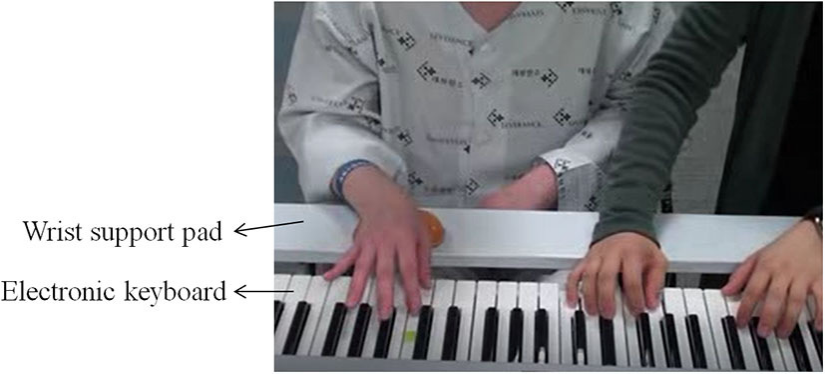
A participant's position for movement-specific keyboard playing intervention.

This study used a musical instrument digital interface (MIDI)-embedded electronic keyboard (DGX-230, Yamaha, Japan) connected to a computer. A MIDI keyboard recorded and computed the velocity of each keystroke, as a measure of key-pressing force, *via* a MIDI sequencing program Cubase 6 (Steinberg Media Technologies AG, Hamburg, Germany). To maximize selective finger control with postural stability and minimize unnecessary compensatory movements in elbow and wrist joints, a wooden wrist support pad that could be attached to the keyboard stand was also used (Figure 1).

### 2.3. Movement-specific keyboard playing

Each participant in the intervention group participated in a 30-min therapeutic keyboard-playing intervention three times a week for 3–4 weeks completing eight to nine intervention sessions. The intervention was conducted individually, in an isolated room at the hospital. Participants sat in front of the keyboard having their upper arms as near as possible to their trunk, elbows flexed at 90 degrees, forearms in the pronation position, and wrists positioned for stable key-striking using extension-flexion finger movements. A wrist support pad was used to help participants maintain their arm position during intervention. The targets of this movement-specific keyboard-playing intervention were individuated and sequential finger movements of the affected hand. The level of movements depended on the number of involved fingers at a time, the configuration of fingers in a sequence, the tempo or force of the key-pressing movement, and the involved hand (unimanual vs. bimanual). Examples of the keyboard-playing tasks used in each of the four levels are displayed in Table 2.

**Table 2:** Target tasks in the movement-specific keyboard playing intervention.

**Level**	**Target keyboard playing task**	**Example**
1	Individuated key-pressing at a self-paced tempo	To depress the key individually, repeating each keystroke three to four times (e.g., F 1 1 1 1–F 2 2 2 2–F 3 3 3 3 …) 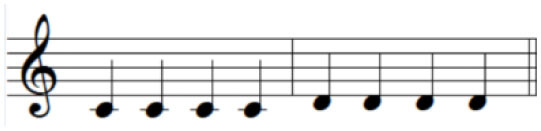
	Simultaneous key-pressing at a self-paced tempo	To depress more than two keys at once using designated fingers 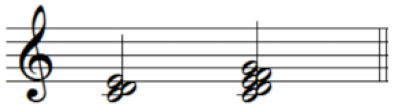
2	Individuated key-pressing with accented notes	To depress the key individually with an increased loudness of specific keystroke (accent) 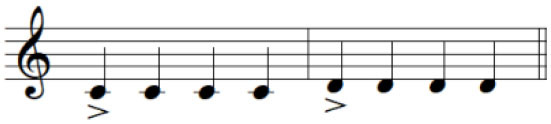
	Sequential key-pressing	To depress more than three keys successively involving five fingers 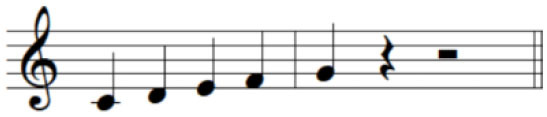
3	Individuated or sequential key-pressing at an increased tempo	To perform the repetitive individuated or sequential key-pressing (level 1 and 2) at an increased tempo (80–100 beats per minute)
	Sequential key-pressing in a random pattern	To depress the five keys sequentially using non-adjacent fingers (e.g., F 1–4–2–5–3) 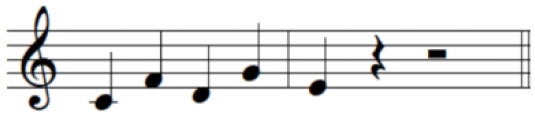
4	Bimanual keyboard playing	To perform the repetitive individuated or sequential playing bimanually 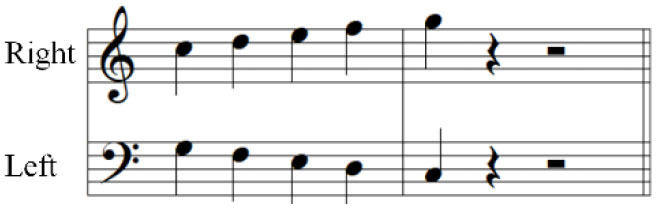

**Figure 2:**
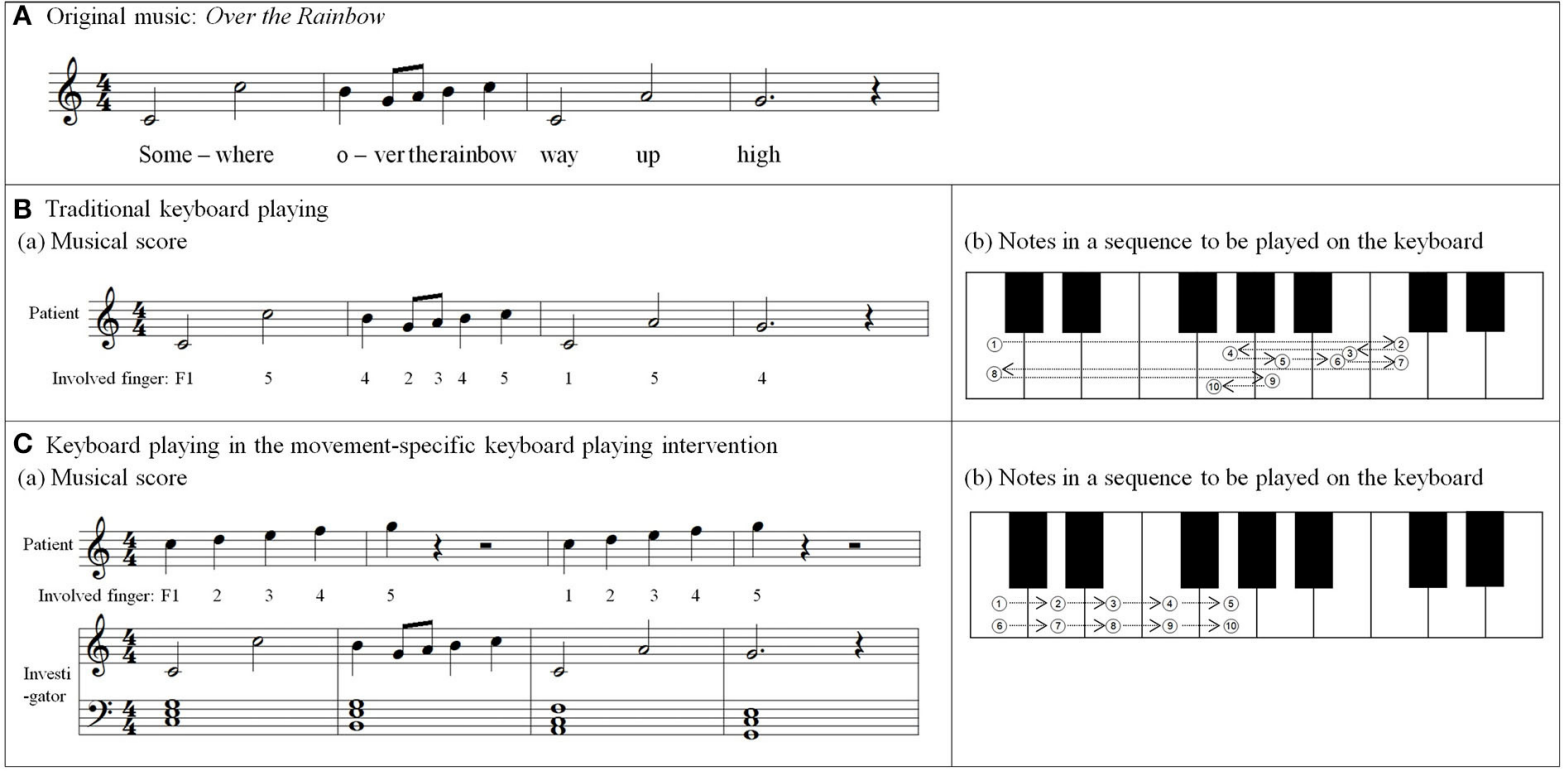
**(A–C)** Structured keyboard playing notes in the movement-specific keyboard playing intervention.

For the initial level, participants performed individuated and simultaneous key-pressing tasks at a self-paced tempo. They depressed five keys individually, repeating each keystroke three or four times. If they showed difficulties in individuated finger movements, participants began with the task of depressing more than two keys at once using designated fingers. For the second level, the applied tasks were individuated and sequential key-pressing movements. For individuated key-pressing tasks, the repetition of key-pressing increased, and the application of accented notes (i.e., increased loudness for a specific keystroke) was required. The primary change in the third level was tempo adjustment. Unlike the former levels in which self-paced tempo or slow tempo was set, participants were asked to perform the key-pressing tasks at an increased tempo (e.g., 80–100 beats per minute). Furthermore, sequential key-pressing in a random pattern was added (sequential movements of non-adjacent fingers such as thumb-ring-index-little-middle fingers, unlike a successive pattern that requires adjacent finger movement in sequential order). For the fourth level, participants engaged in bimanual keyboard playing using both affected and unaffected (or less affected) hands. They were asked to perform the repetitive individuated and sequential key-pressing tasks that were applied in the former levels while using both hands (Figure 2).

### 2.4. Measures

This study measured the FIM that assesses an individual's independence or degree of disability in relation to performing daily activities timely and safely. It consists of 18 items in total and includes four subscales: 13 items for motor function (self-care, sphincter control, and mobility) and five items for cognitive function (communication and social cognition). Each item is scored from 1 (needing total assistance) to 7 (performing a task with complete independence) and the total score ranges from 1 to 126. The self-care subscales included the independence of task performance during the six activities (i.e., feeding, grooming, bathing, dressing upper body, dressing lower body, and toileting). In this study, each participant was measured by an assessor who was blind to their group assignment.

A key-pressing test and hand function tests were also administered before and after the intervention. The key-pressing test was implemented by an investigator using a MIDI keyboard. Each participant was asked to individually depress five keys involving five fingers as strong as possible in two patterns: Fingers 1-2-3-4-5 and Fingers 5-4-3-2-1. Each task was repeated three times with a rest between trials, and the measured velocity from each trial was averaged. Additionally, certified occupational therapists, who were blind to group assignment, implemented three-hand function tests: grip and pinch power, BBT, and the Jebsen-Taylor Hand Function Test (JTHFT). The grip and pinch power test measured the maximal force when participants held a handle of the dynamometer (grip) and held the pinch gauge while placing it between the tip of the thumb and the tip of the index finger (tip pinch), between the pad of the thumb and lateral surface of the index finger (lateral pinch), and between the pad of the thumb to the pads of the index and middle fingers (palmar pinch). The BBT was administered using a box divided into two compartments containing 150 cubes. During the test, participants moved cubes, one by one, from one compartment of the box to the other, within 1 min. The number of cubes moved is indicative of the level of manual dexterity. The JTHFT consists of seven subtests: writing, turning a card, picking up small objects and placing them in a container, stacking checkers, simulating feeding, moving light objects, and moving heavy objects. The time to complete each test was measured, and a shorter time is indicative of greater manual function.

### 2.5. Data analysis

For each measure, the mean and standard deviation was calculated. In addition, a mixed model of repeated measures ANOVA was conducted to examine the group differences in such hand function-related parameters at two different time points (pre- and post-test).

## 3. Results

The results of a mixed model of repeated measures ANOVA are displayed in Table 3. There were significant time effects in most of the measured parameters, except in key-pressing forces in the index, middle, and ring fingers, and the time to complete a simulated eating task after rehabilitative training. This indicates that rehabilitative training, either standard care or movement-specific keyboard playing added to standard care, enhanced hand function of the affected side in individuals.

**Table 3:** Comparison of changes in hand function-related parameters between groups.

	**Intervention group (*****n*** = **12)**		**Control group (*****n*** = **11)**		**Time**		**Group**		**Time** ^*^Group	
**Parameter**	**Pre-test** ***M*** ±***SD***	**Post-test** ***M*** ±***SD***	**Pre-test** ***M*** ±***SD***	**Post-test** ***M*** ±***SD***	*F*_(1, 21)_	***p***	*F*_(1, 21)_	***p***	*F*_(1, 21)_	***p***
**FIM, score**										
Total	91.0 ± 20.3	101.2 ± 13.8	96.5 ± 15.5	103.0 ± 14.4	13.271	0.002^**^	0.325	0.575	0.623	0.439
Self-care subscale	26.5 ± 8.2	31.8 ± 5.4	29.7 ± 7.0	33.0 ± 5.9	15.368	0.001^**^	0.722	0.405	0.881	0.359
**Key-pressing force, velocity (Range 0–127)**										
Thumb	36.6 ± 21.1	52.3 ± 25.8	40.1 ± 20.8	51.2 ± 34.6	7.477	0.012^*^	0.016	0.902	0.220	0.644
Index finger	36.7 ± 10.0	44.0 ± 16.8	46.3 ± 16.6	48.0 ± 32.2	1.079	0.311	0.875	0.360	0.413	0.527
Middle finger	39.3 ± 17.6	46.8 ± 17.0	50.3 ± 21.2	50.7 ± 27.1	0.935	0.345	0.929	0.346	0.749	0.396
Ring finger	45.9 ± 19.5	51.1 ± 14.4	54.1 ± 18.8	54.0 ± 29.4	0.308	0.585	0.545	0.469	0.353	0.559
Little finger	39.1 ± 18.8	50.4 ± 16.2	47.8 ± 20.8	58.9 ± 27.8	13.121	0.002^**^	1.081	0.310	0.001	0.975
Grip power, kg	8.6 ± 9.8	11.4 ± 9.3	18.9 ± 16.0	23.5 ± 25.0	6.345	0.020^*^	2.732	0.114	0.387	0.541
Tip pinch, kg	2.2 ± 2.2	2.8 ± 2.1	3.7 ± 3.7	4.5 ± 4.3	7.923	0.010^*^	1.605	0.219	0.122	0.730
Lateral pinch, kg	3.6 ± 2.4	4.3 ± 2.0	4.4 ± 1.8	5.3 ± 2.3	9.262	0.006^**^	1.178	0.290	0.244	0.627
Palmar pinch, kg	3.0 ± 2.5	4.1 ± 2.3	3.6 ± 1.1	4.4 ± 1.8	13.613	0.001^**^	0.306	0.586	0.317	0.580
BBT, numbers/min	31.6 ± 17.6	37.5 ± 15.0	32.2 ± 17.7	36.2 ± 19.4	16.305	0.001^**^	0.002	0.965	0.609	0.444
**JTHFT, seconds**										
Writing	88.1 ± 61.6	48.7 ± 31.6	91.3 ± 127.0	47.5 ± 33.6	4.411	0.048^*^	0.002	0.966	0.012	0.914
Card turning	18.6 ± 14.9	10.8 ± 7.7	12.6 ± 7.4	11.1 ± 7.1	5.693	0.027^*^	0.598	0.448	2.489	0.130
Small object moving	23.5 ± 17.0	17.1 ± 9.0	17.1 ± 15.4	10.5 ± 5.0	7.264	0.014^*^	1.913	0.181	0.001	0.977
Simulated eating	61.6 ± 123.5	54.6 ± 125.2	18.5 ± 13.2	60.0 ±137.7	0.759	0.394	0.182	0.674	1.496	0.235
Checker stacking	30.0 ± 20.7	20.7 ± 17.4	12.7 ± 6.4	11.3 ± 7.0	8.596	0.008^**^	4.617	0.043^*^	4.678	0.042^*^
Heavy can lifting	11.1 ± 9.3	7.7 ± 4.7	7.8 ± 4.2	6.0 ± 2.2	7.068	0.015^*^	1.150	0.296	0.665	0.424
Light can lifting	10.8 ± 7.2	6.8 ± 4.0	7.5± 4.1	5.9 ± 2.3	15.319	0.001^**^	1.255	0.275	2.945	0.101

Data presented as M ± SD. FIM, Functional independence measure; BBT, Box and block test; JTHFT, Jebsen-Talyor hand function test.

The score of the self-care subscale ranges to 7–42 with higher scores indicating increased independence in performing self-care tasks.

* *p* < 0.05.

** *p* < 0.01.

**Figure 3:**
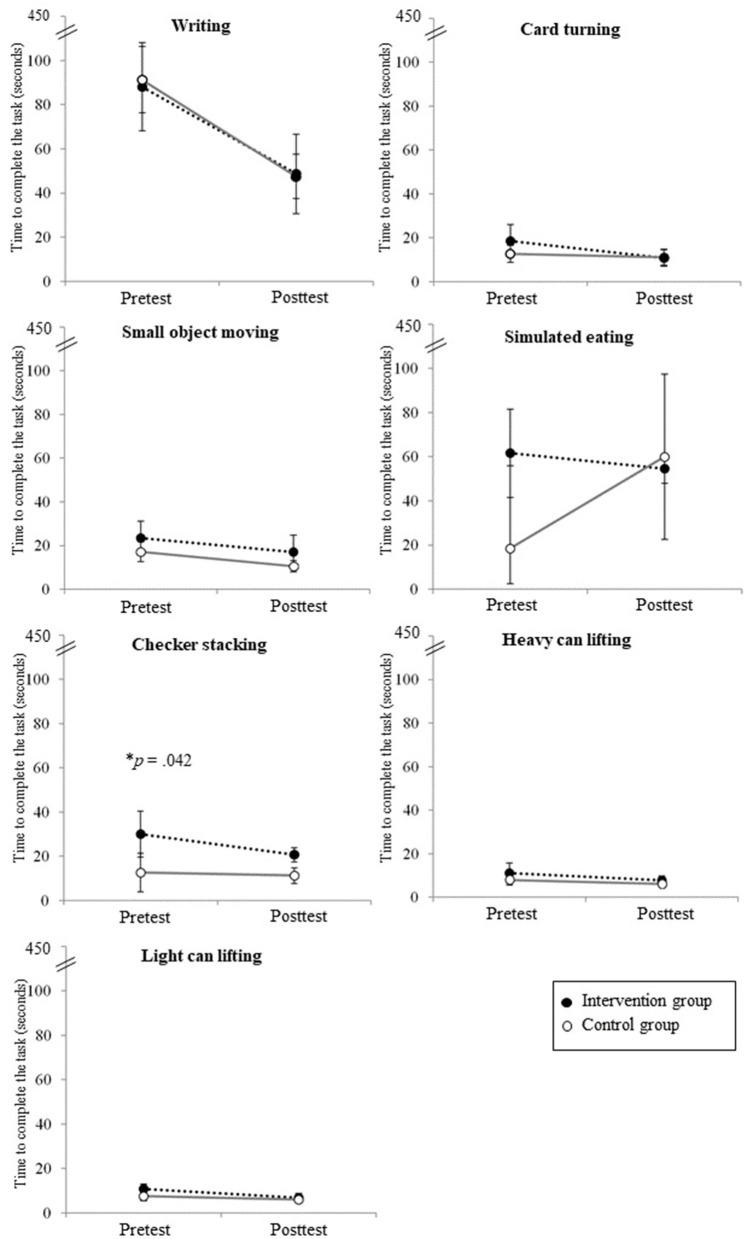
The changes in seven subtests of the JTHFT in each group. The interaction effect between group and time was significant only in the checker-stacking test.

The group effect and the interaction effect between time and group were significant only in the task of checker stacking as a subtest of the JTHFT. For the checker-stacking task, the intervention group showed a greater decrease in the time required to complete the task than the control group (Figure 3), which reaches a statistical significance. Although the trend of change was not significantly different between the groups except in the checker-stacking task, changes in other subtests of the JTHFT demonstrated the intervention group showed a tendency to decrease at a greater level of all the task completion time. However, the control group showed smaller changes of the completion time in general, and even an increase of the time in simulated eating.

## 4. Discussion

This study aimed to examine how movement-specific keyboard playing enhanced fine motor functions in adolescents and young adults with acquired brain injury by comparing their outcomes with those of individuals who received only standard care without such keyboard playing. A mixed model of repeated measures ANOVA showed that significant time effects were found in most of the measures. This supports that a rehabilitative training that intensively involves fine motor skills contributed to improvements in the upper limb movements required to perform activities of daily living. Given that the self-care subscale measures functional abilities in bathing, dressing the upper body, dressing the lower body, eating, grooming, and toileting, the slightly greater increase in the self-care subscale in the intervention group might be supported by previous studies demonstrating that the intensive practice of finger movements affects functional changes (11, 12), as well as neurological plasticity through reorganizing interregional connectivity (16).

In terms of key-pressing force, the intervention group showed increased values in more fingers compared to the control group. For keyboard playing, finger force is associated with speed and the acceleration of movements to strike the keys (17). Key-pressing movements recruit forearm muscles that control forearm movements, both involving individuated and synergic movements (11). This indicates that increased key-pressing forces are related to increased and more focused muscle activation. Although insignificant group differences may limit conclusive implication, greater increases in finger forces observed after the intervention suggest that movement-specific keyboard playing may involve individuated finger movements intensively and efficiently.

Both groups showed a similar level of grip and pinch power and BBT. It is noteworthy that a significant interaction effect of group and time was found in the checker-stacking task, one of the JTHFT subtests. Although the control group, who received only standard care, also showed improvement in finger strength and grasping movements, the intervention group showed significant changes in tasks requiring more precise and dexterous manipulation with the hands and fingers. As an unpracticed task, checker stacking involves eye-hand coordination, which is represented in keyboard playing involving visually guided reaching and striking finger movements. In this study, movement-specific keyboard playing involved individuated, sequential, and simultaneous movements across fingers, while coordinating the timing and forces of each movement (18). While standard training for fine motor skills is more focused on synergic finger movements required to perform a task and primarily requires the ability to manipulate objects, movement-specific keyboard playing involves individuated motion of each finger. Deliberate practice of each finger movement with temporal constraint and auditory and tactile feedback specific to each executed movement intervenes with more selective motor control of fingers (7). Furthermore, engagement in more precise and dexterous finger movements during keyboard playing has been associated with the ability to manipulate objects requiring more sophisticated and complex movements (19).

The greater changes documented in the intervention group may be associated not only with the massive practice of finger movement but also with the structured intervention for individuated finger movement. This study incorporated movement-specific keyboard playing into a structured intervention while adjusting the type and tempo of the key-pressing movements. When executing movements in a musical environment, direct auditory feedback is beneficial for providing information on the timing and quality of the target movements, which helps patients plan, execute, and modify their finger movements within temporal and spatial constraints (17, 20). The pronounced findings in this study support that such keyboard playing may be an option for increasing the expected intervention outcomes.

However, the generalization of the results should be cautious in consideration of the differences in hours of training between groups. The intervention group received more hours of training as a result of the addition of movement-specific keyboard playing to standard care. Further studies that control the types and hours of training received and have an increased sample size are needed to confirm that the observed differences between the groups are attributed to the specified movements applied.

Movement-specific keyboard playing has the potential of optimizing the auditory and musical feedback from engagement in movement-specific keyboard playing, which may enhance the benefits of goal-directed rehabilitation. Finger movements are fine motor function, and accomplishment of the movements may be perceived in correlation with the quality of the movement and corresponding auditory feedback. Various musical outputs produced in this study might enhance young patients' motivation for rehabilitative training and compliance with structured programs, thus leading to desired changes in fine motor skills. However, the ability to generalize the results without further measuring perception and motivation in patients is limited. In addition, the lack of direct comparison between the different types of auditory feedback in this study should be cautiously considered.

In conclusion, this study supports that movement-specific keyboard playing may be a potential option for appropriate rehabilitative intervention for adolescents and young adults with acquired brain injury. This study also corroborated that the specified finger movements in a sequence led to dexterous and more controlled selective movements of the hands and fingers required for performing tasks during daily activities. Additional studies are needed to investigate the mechanism for differential auditory feedback during keyboard playing. Along with clinical improvement, the maintenance of intervention outcomes will be assessed in further studies with a follow-up test.

## Data availability statement

The raw data supporting the conclusions of this article will be made available by the authors, without undue reservation.

## Ethics statement

The studies involving human participants were reviewed and approved by College of Medicine, Yonsei University. Written informed consent to participate in this study was provided by the participants' legal guardian/next of kin.

## Author contributions

SK and S-RC designed the study, reviewed and approved the manuscript, and also were responsible for the integrity of the data as co-corresponding authors. Y-KS and EJ contributed to the acquisition and interpretation of the data and writing of the manuscript. All authors made substantial contributions to this study. All authors contributed to the article and approved the submitted version.
